# Examination for Meat-Inspector, October 22, 1901

**Published:** 1901-10

**Authors:** 


### COMMUNICATIONS.

### EXAMINATION FOR MEAT-INSPECTOR, OCTOBER 22, 1901.

The United States Civil-service Commission invites attention to the examinations which will be held at various places throughout the United States on October 22d for the position of meat-inspector.

During the past three years, in addition to the regular semi-annual, a number of special examinations have been held for this position; but the Commission has failed to secure a sufficient number of eligibles to meet the need of the service, every person who has passed the examination having been offered an appointment. This examination offers a most excellent opportunity to secure employment in the Government service at a salary of from $1200 to $1400 per annum.

Information relative to the scope of the examination may be found in Section 60 of the *Manual of Examinations*, revised to January 1, 1901.

Age limit, twenty years or over.

From the eligibles resulting from this examination it is expected that certification will be made to the position of meat-inspector in the Bureau of Animal Industry, Department of Agriculture.

This examination is open to all citizens of the United States who comply with the requirements and desire to enter the service. All such persons are invited to apply, and applicants will be examined, graded, and certified with entire impartiality and wholly without regard to any consideration save their ability as shown by the grade attained in the examination. Preference in certification may be given to eligibles who are legal residents of the place or vicinity where the vacancy exists.

Persons who desire to compete should at once apply to the United States Civil-service Commission, Washington, D. C., for a copy of the *Manual of Examinations* and Application Forms 304 and 375. The application should be properly executed and promptly forwarded to the Commission.

All persons who have been examined for this position during the past year and failed to pass will be allowed re-examination upon filing a new application.

October 3, 1901.

